# The evolutionary significance of the earliest cloacal opening in Synapsida

**DOI:** 10.1016/j.isci.2026.116752

**Published:** 2026-07-10

**Authors:** Lorenzo Marchetti, Antoine Logghe, Arnaud Rebillard, Mark J. MacDougall, Jörg Fröbisch

**Affiliations:** 1 Museum für Naturkunde Berlin, Leibniz-Institut für Evolutions- und Biodiversitätsforschung, Invalidenstraße 43, Berlin 10115, Germany; 2 Centre de Recherche en Paléontologie – Paris, UMR 7207 – CNRS, MNHN, SU, Muséum National d’Histoire Naturelle, 8 rue Buffon, CP38, Paris, France; 3 Institut für Biologie, Humboldt-Universität zu Berlin, Invalidenstraße 42, Berlin 10115, Germany; 4 Brandon University, 270 18th Street, Brandon, MB R7A 6A9, Canada

**Keywords:** cloaca, vent, resting trace, synapsids, trace fossils, early Permian, evolution, Bromacker, terrestrial

## Abstract

The cloaca is a fundamental structure of modern terrestrial vertebrates, including the terminal parts of the urogenital and digestive systems that converge in a single channel and opening (vent). Although it is widespread in amphibians and reptiles (including birds) and occurs in mammals, the fossil record of this structure is scarce. Here we present an exceptional specimen from the early Permian Bromacker locality (294 Ma), which includes a tail impression with two rows of elevated scales separated by a vertical slit, that we interpret as cloacal lips and vent. Because of its morphology and association with the footprint *Dimetropus*, we attribute this impression to caseid synapsids. This represents the first occurrence of a cloaca in the fossil record of stem mammals. The comparison with modern and fossil specimens allows us to hypothesize a vertical vent orientation for early crown tetrapods, that subsequently changed orientation at least three times during tetrapod evolution.

## Highlights

• A 294 Ma tail impression with epidermal scales and cloacal opening is described
• This impression is associated with *Dimetropus* and attributed to caseid synapsids
• The cloacal opening (vent) is sub-vertical as in modern amphibians and crocodiles
• The orientation of the vent changed at least three times during tetrapod evolution

## Graphical abstract

**Figure:**
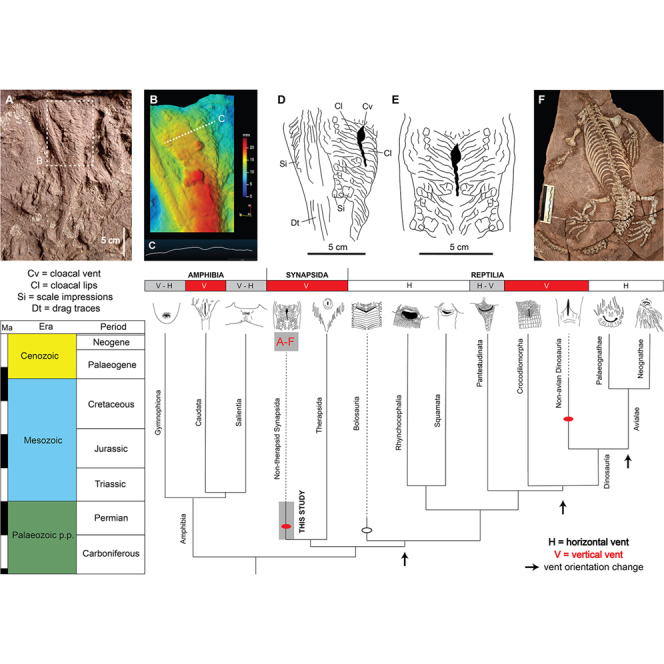

## Teaser

Zoology; evolutionary biology; Paleobiology

## Introduction

The cloaca is the enlarged, final section of the hindgut, found in many modern terrestrial vertebrates,^1^ such as amphibians, reptiles (including birds) and, more rarely, mammals. It comprises the terminal parts of the urogenital and digestive systems, which converge into a single channel with a single opening (or vent) in the ventral part of the animal,^2^ commonly placed in the medial part of the anterior region of the tail.^1^ This configuration enables a wide range of biological (e.g., protection, excretion, reproduction, oviposition, thermoregulation, gas exchange, water regulation) and behavioral (e.g., mating, visual signaling, chemical communication) functions. The fossil record of this body orifice is of primary importance to better understand the physiology of extinct animals and the evolutionary history of this structure. However, the fossil record of cloacae is scarce due to the rarity of soft tissue preservation.^3^ Trace fossils and in particular resting traces, showing the anterior part of the tail, can provide valuable information on the morphology and orientation of the externally visible part of cloacae.^4^ Here we describe the first and earliest evidence of this structure in the fossil record of Synapsida, the mammalian lineage of amniotes. Our study highlights a vertical orientation of the vent in early synapsids of 294 Ma,^5^ whereas a horizontal vent orientation was recently observed in early reptiles of 298 Ma.^4^ We hypothesize a vertical vent orientation as plesiomorphic for crown tetrapods. Hence, the morphological transition to a horizontal vent already occurred among amniotes in the late Paleozoic. Moreover, we identify at least three different changes of vent orientation in the evolutionary history of tetrapods: (1) from sub-vertical (early synapsids) to horizontal (stem reptiles) in the late Paleozoic; (2) from horizontal to vertical (non-avian archosauriforms) in the Triassic, and (3) from vertical to horizontal (avian dinosaurs) in the late Mesozoic. Nevertheless, the reasons for these morphological changes remain unclear.

## Results

### Specimen description

**Figure 1:**
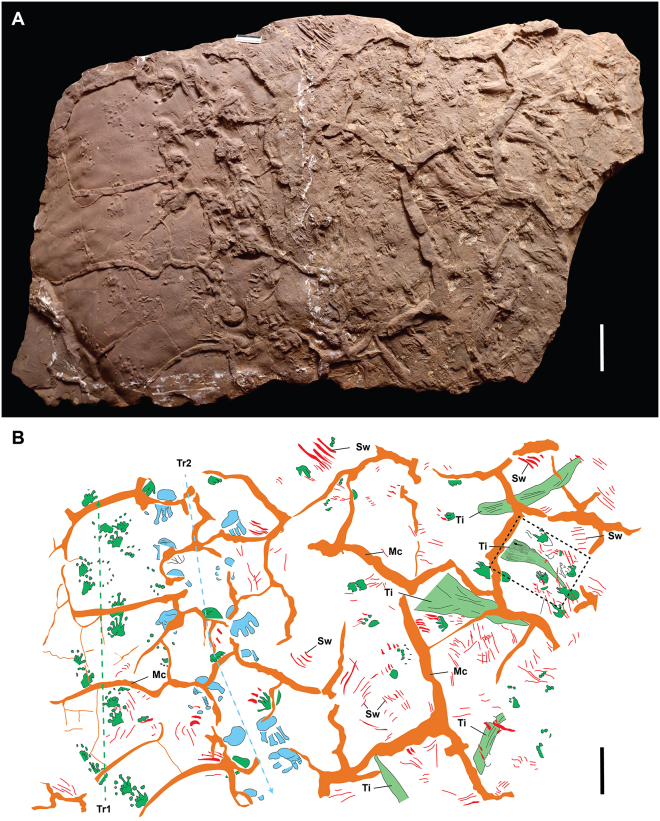
The studied specimen (A) MNG 13490, convex hyporelief. (B) Interpretive drawing. Tr1, trackway of *Dimetropus leisnerianus*; Tr2, trackway of *Ichniotherium sphaerodactylum*; Ti, tail impression; Sw, swimming trace; Mc, mud crack. Scale bars, 20 cm.

**Figure 2:**
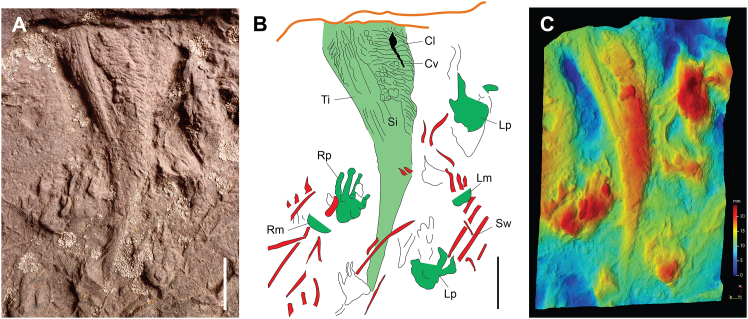
The analyzed tail impression and cloacal area (A) Tail impression (Ti) with epidermal scale impressions (Si), a vertical cloacal vent (Cv) and cloacal lips (Cl), associated with *Dimetropus* isp. footprints arranged in a step cycle (p, pes; m, manus; L, left; R, right) and Sw, swimming traces. Convex hyporelief. (B) Interpretive drawing. (C) False-color depth map. Scale bars, 5 cm.

Specimen MNG 13490 (Figure 1) shows several vertebrate trace fossils preserved as natural casts, including tetrapod footprints (*Dimetropus*, *Ichniotherium*, *Varanopus*), as well as tetrapod swimming traces.^5^ On the right side of the specimen, a few so far undescribed, elongated and tapering impressions are observed, here interpreted as tetrapod tail traces. One of these impressions (Figure 2) is associated with elongated footprints, arranged in a step cycle that does not overlap the tail impression. The direction of the tail impression is congruent with the direction of the step cycle.^6^ Also, the tail impression is placed roughly in the middle of the step cycle (Figure 2D). Four consecutive pedal footprints are recognized (Figure 1), and their size, morphology, and arrangement strongly support the association with this tail impression. The best preserved of these footprints (right pes) is about 54 mm long. This footprint, preserving digits I, III, IV, and V has a long sole impression and relatively thin digit imprints, increasing in length from I to IV, with well-impressed circular basal pads, features characteristic of *Dimetropus*.^5^ The tail impression is about 264 mm long and 81 mm wide in its anteriormost part, is tapering distally, and is cut by a mud crack anteriorly. It is also deeper and slightly curves distally toward its right side. This apparent asymmetry only allows us to observe the right lateral part of the anterior tail impression. A groove observed on its right side is suggestive of drag movement (Figure 2), which is in agreement with a stop and re-start of locomotion.^6^

### Cloacal area

**Figure 3:**
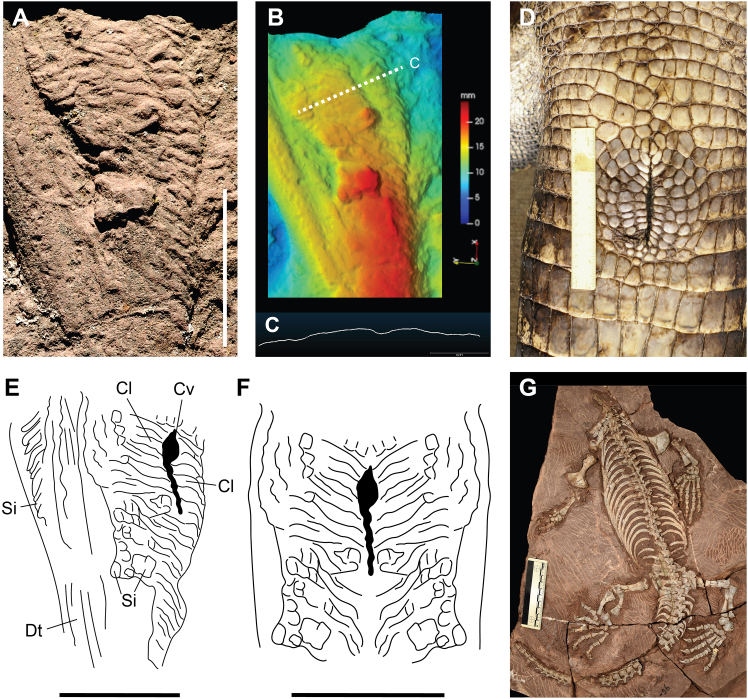
The cloacal area and the potential producer (A) Close-up of the cloacal area of MNG 13490, convex hyporelief. (B) False-color depth map. (C) 3D model vertical cross-section. (D) Vertical cloacal vent and scalation pattern in a modern crocodile. From Vinther et al.^3^ (E) Interpretive drawing of the cloacal area, ventrolateral view. Epidermal scale impressions (Si), vertical cloacal vent (Cv), cloacal lips (Cl), drag traces (Dt). (F) Reconstruction of the cloacal area, ventral view. (G) Potential tracemaker. MNG 14230. *Martensius bromackerensis*, paratype, complete skeleton in dorsal view, juvenile. Scale bars, 5 cm.

The central part and left side of the tail impression of MNG 13490 (Figure 3A) show two clusters of elevated and elongated polygonal structures arranged in anteriorly convex rows. We interpret these structures as epidermal scales, based on their homogeneous morphology and arrangement.^4^^,^^5^ As they only occur on the resting trace and have a homogeneous arrangement, we disregard these impressions as potential microbially induced sedimentary structures (MISS). The two rows of scales are divided by a clear vertical discontinuity that is about 37 mm long and 7 mm wide (Figures 3E and 3F). This discontinuity is clearly a depression, as evidenced by the section of the 3D model (Figures 3B and 3C). The anterior and central placement of the two rows of scales within the ventral tail impression, their elongation and their separation by a vertical slit, contrast other scale impressions on the tail (which are laterally tapered or shortened). Furthermore, the shape of these rows of scales is not disrupted by the mud crack and the tail impression curvature.

We interpret these elevated rows of scales as the lateral lips of a cloaca, with the vertical discontinuity being the vent, because they are reminiscent of cloacal openings in modern and fossil tetrapods^3^^,^^4^ (Figure 3D and Table S1). Indeed, cloacal openings are normally placed antero-centrally in the ventral part of the tail.^2^ Moreover, they are commonly characterized by an elongated opening (vent) surrounded by two elevated areas (lips), presenting scale morphology that differs from other ventral scales (either smaller or larger, and with a different arrangement).^4^

## Discussion

### Trace-tracemaker correlation

As *Dimetropus* has been consistently attributed to early diverging synapsids (except for varanopids),^5^^,^^7^ we also attribute the associated tail impression of MNG 13490 to this group. This includes caseasaurs, ophiacodontids, edaphosaurids, and sphenacodontians. The pes and tail impression length, as well as the tail impression tapering aspect, are consistent with MNG 14230, a juvenile specimen of the caseid caseasaur *Martensius bromackerensis* from the same locality (Figure 3G).^5^ In fact, the tail length of this specimen is about 359 mm and the pes length (measured parallel to the metatarsal III and excluding the tarsus) is about 77 mm. The ratio tail length/pes length is 4.65, which is similar to the same ratio measured in the trace (4.87). Moreover, the described tail impression clearly differs from the recently described sphenacodontid tail impressions *Bromackerichnus requiescens*, which are thinner, not strongly tapering and with more prominent and laterally narrow scales.^5^ Also, ophiacodontids and edaphosaurids are currently not known from the Bromacker locality,^5^ despite intensive sampling. Therefore, a caseid synapsid producer is most consistent with the described specimen.

### Evolutionary significance

**Figure 4:**
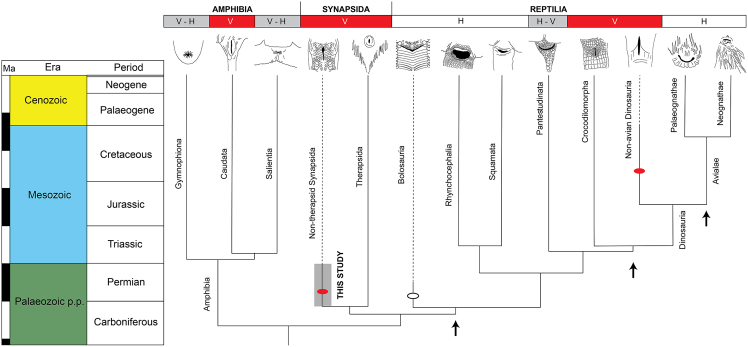
Cloacal structure evolution Phylogenetic scheme with the main present-day tetrapod group cloacal vents and the fossil occurrences (ellipses). V, vertical vent orientation (red color); H, horizontal vent orientation (white); intermediate status (V-H or H-V) in gray. The arrows indicate in which position of the tree the cloacal vent orientation changed.

The hereby described trace fossil attributed to a caseid producer represents the earliest and thus far only evidence for a cloacal opening in the fossil record of Synapsida. Morphologically, it is similar to vertically oriented vents observed in present-day salamanders, monotreme mammals, and crocodiles^3^ (Figure 4 and Table S1). It differs from the typical squamate^3^ and early reptile^4^ sub-horizontal vents (Figure 4). Due to occurring in early diverging synapsids (Caseidae; this work) and in some of the basal-most families of extant amphibians (Ichthyophiidae, Cryptobranchidae, Ascaphidae; Table S1), vertical vent orientation was likely the plesiomorphic condition for crown tetrapods. So, the horizontal orientation observed in some modern families of amphibians likely developed after the Triassic (Figure 4). The evolutionary transition from a vertical vent of crown tetrapods and early synapsids (this work, about 294 Ma) to a horizontal vent orientation most likely happened in late Paleozoic representatives of the reptilian lineage, with a first documented occurrence of a horizontal vent in the early Asselian (about 298 Ma) bolosaurian traces *Cabarzichnus pulchrus*^4^ (Figure 4). More derived archosaurian reptiles, such as crocodilians and non-avian dinosaurs, exhibit a reversal to a vertical vent, as evidenced by the Early Cretaceous ornithischian dinosaur *Psittacosaurus*.^8^ However, it should be noted that other authors left the interpretation of the cloacal vent orientation of *Psittacosaurus* open.^3^ Considering the timing of the Crocodylomorpha appearance, this orientation change likely happened during the Triassic (Figure 4). Some modern turtles show a vertical vent, this is likely a derived feature not present in basal forms, because the basal-most turtle families show a horizontal vent orientation (Figure 4 and Table S1). A predominantly horizontal arrangement is observed in present-day birds (Figure 4), probably indicating an orientation shift among saurischian dinosaurs later in the Mesozoic.

**Figure 5:**
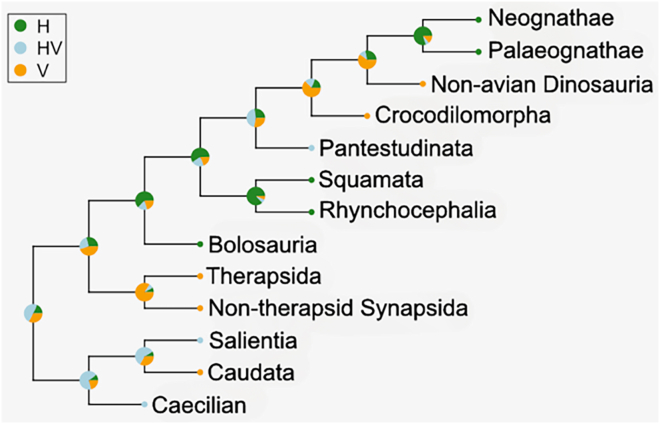
Cloacal structure ancestral state reconstruction Phylogeny presented in Figure 4 with ancestral states reconstructed at nodes. V, vertical vent orientation; H, horizontal vent orientation; HV, intermediate status.

Ancestral state reconstruction using the three potential cloacal orientations supports the inference that a vertical orientation is most likely the plesiomorphic state for synapsids and a horizontal orientation is most likely the plesiomorphic state for reptiles (Figure 5).

Therefore, the orientation of the vent changed multiple times (at least three) during the evolutionary history of terrestrial vertebrates. A possible reason for this is a different morphology of the reproductive organs, in particular the occurrence or not of a phallus (penis or hemipenis^9^^,^^10^). These changes in the vent morphology might also have been linked to other biological functions of the cloacal vent such as in squamates and birds, where cloacal evaporation for thermoregulation purposes has been observed.^11^ Other possible explanations involve: sexual display (e.g., in birds)^12^; defensive purposes by secretions or sounds (e.g., in snakes)^13^; respiration (in aquatic turtles)^14^; urination/defecation and laying eggs, which may require different anatomical features based on the lifestyle and paleoenvironment of the animal (Table S1). Finally, the analysis of our dataset suggests a possible link between the length and orientation of the cloacal vent and the reduction or loss of the tail or limbs (e.g., caecilians, anurans, turtles) (Figure 4 and Table S1).

To our knowledge, there have been no studies dealing specifically with the different orientation of the cloacal vent and its function. Nevertheless, we can advance some preliminary hypotheses. We notice that both squamates and birds, in which cloacal evaporation for thermoregulation purposes has been observed,^11^ show an horizontal cloacal orientation. Conversely, groups that have a sub-vertical orientation such as Caudata and Crocodylomorpha, are tied to aquatic environments. Also, the tail impression of MNG 13490 is clearly impressed in a water-saturated sediment, and the same specimen shows swimming traces associated with *Dimetropus* footprints and overlapping the tail trace (Figure 2B). So, it is possible that a transition from a vertical to an horizontal vent would favor an adaptation to drier environments by cloacal evaporation. This would be in agreement with the vent orientation change in stem reptiles between the Carboniferous and the Permian, in a time of global warming and increasing aridity and seasonality.^4^ Nevertheless, the causes of this correlation should be further investigated in modern taxa.

A variable occurrence and morphology of the phallus is observed within Squamata,^9^ but this does not modify vent orientation. All the described groups (Figure 4 and Table S1) lay eggs, and the occurrence of soft or hard shells does not seem to vary with the vent orientation. Other possible explanations such as sexual display, defensive purposes, and cloacal respiration seem to be limited to a few taxa and not to apply to higher level groups (Table S1). The reduction of tail and limbs seem to favor shorter cloacal vents and a more variable vent orientation within the group (Table S1). This might be related to the necessity of protecting the cloacal opening without the cover of a long tail and limbs.

Although the reasons for varying vent orientations are diverse and difficult to discern especially in a scarce fossil record, the earliest synapsid cloacal opening provides unique insights into its evolution in early amniotes.

### Limitations of the study

Our description and interpretation are based on a single resting trace, which is incomplete because it is anteriorly cut by a mud crack. Also, a groove on its right side might indicate tail dragging. This is why, for the time being, we refrain to introduce a separate ichnotaxonomy for this tail impression, although clearly different from other synapsid tail impressions (*Bromackerichnus requiescens*).^5^ Another limiting factor is the low resolution of the track-trackmaker attribution of the associated trace *Dimetropus*. This ichnogenus is generally attributed to a broad range of synapsid families^5^ including caseasaurs, ophiacodontids, edaphosaurids, and sphenacodontians. Nevertheless, our evolutionary discussion relies on broader groups (e.g., non-therapsid synapsids) so a more precise track-trackmaker correlation would not be needed. Also, additional trace-tracemaker correlations were made based on the tail impression alone. The fact that this is a trace fossil implies that only the external morphology is registered in the sediment, which constrains the study to the evolution of the external surface of the cloacal area. The conditions of preservation of the specimen, which is impressed on its right ventrolateral side and the presence of a tail impression curvature, oblige to reconstruct the original symmetry of the ventral side, which was done in Figure 3F. The limited occurrence of fossils displaying the external surface of the cloacal area^3^^,^^4^ is a limiting factor regarding a detailed understanding of the deep-time evolution of cloacal morphology and orientation, especially in long-extinct lineages. Nevertheless, this is implemented with a large database representing a large diversity of modern terrestrial tetrapods (Table S1), providing a robust phylogenetic constraint for considerations on cloacal evolution.

## Resource availability

### Lead contact

Further information and requests for resources and reagents should be directed to and will be fulfilled by the lead contact Dr. Lorenzo Marchetti (lorenzo.marchetti@mfn.berlin).

### Materials availability

The authors declare that the specimen MNG 13490, which is the focus of this study, is housed at the Friedenstein Stiftung Gotha (MNG), Germany.

### Data and code availability

• The studied material is available at publicly accessible institutions. Institutional abbreviations: MNG, Friedenstein Stiftung Gotha, Germany.
• This paper does not report original code.
• Additional information: Any additional information required to reanalyze the data reported in this paper is available from the lead contact upon request.

## Acknowledgments

We thank the editor O. Brusa, the reviewers J. Vinther, S. Voigt, M. Pittman, and an anonymous reviewer for the fruitful discussion. We thank T. Hübner and S. König (both MNG) for the assistance during study visits. We thank all curators and collection managers of other institution with comparative material. This work has been funded by the German Federal Ministry of Research, Technology and Space (BMFTR, formerly 10.13039/501100002347BMBF) for the BROMACKER project 2020–2025, grant number 01UO2002A. A.L. was funded by the Humboldt Research Track Scholarship (2021–2022) and the Interface Pour le Vivant (10.13039/501100019125Sorbonne Université, 2022–2025) during this study. A.R. was funded by an Elsa-Neumann scholarship (State Berlin, Germany).

## Author contributions

L.M. ideated the study, studied the material, made measurements, analyzed data, wrote the manuscript and made figures and tables; M.J.M. analyzed data, made figures, and wrote the manuscript; A.L., A.R., and J.F. analyzed data and wrote the manuscript. All authors revised the manuscript.

## Declaration of interests

The authors declare no competing interests.

## STAR★Methods

### Key resources table

**Table:** 

REAGENT or RESOURCE	SOURCE	IDENTIFIER
**Deposited data**		

Cloaca external morphology	This paper	Table S1

**Software and algorithms**		

Agisoft Metashape Professional	https://www.agisoft.com/	N/A
Cloud Compare	https://www.danielgm.net/cc/	N/A
Paraview	https://www.paraview.org/	N/A
R	https://www.r-project.org/	N/A
phytools	https://besjournals.onlinelibrary.wiley.com/doi/10.1111/j.2041-210X.2011.00169.x	N/A

**Other**		

Described specimen	This paper	MNG 13490

### Experimental model and study participant details

#### Specimen collection

The studied material, the specimen MNG 13490 (Figure 1), was found at the Bromacker locality (Tambach Formation, Thuringia, Germany, about 294 Ma),^15^^,^^16^ coming from tabular sandstone layers (the lower beds of Eberth et al.^17^ and the unit BRO III of Marchetti et al.^16^) and accessioned in 2004.

### Method details

#### Biostratigraphy

All the material from the Bromacker locality belongs to the Tambach Sandstone Member of the Tambach Formation, most recently considered of latest Asselian age^16^ The paleoenvironment has been interpreted as an inland fluvial setting in a seasonal tropical climate.^18^^,^^19^

#### 3D model generation

We obtained 3D models of the specimens through digital photogrammetry^20^ with the software Agisoft Metashape Professional. Contour lines and color depth maps were obtained by employing the software Cloud Compare and Paraview.

#### Taphonomy

The taphonomy of skin impressions has been carefully evaluated, excluding skin-like sedimentary structures such as interference ripples and microbially induced sedimentary structures.^5^^,^^21^ The attribution of skin structures to epidermal scales and cloacal areas are based on morphological comparison with modern-day reptile epidermal scales^22^ and cloacal areas^3^ (Table S1). The scalation pattern and associated structures are described and compared with the fossil record of skin structures including other body impressions,^4^^,^^5^ as well as neontological data. This allows for discussion of the potential phylogenetic placement of the producer and its implications for the evolution of the scalation pattern and related structures in early amniotes.

#### Tracemaker attribution

The attribution to the potential tracemaker is based on a comparison of the morphology and proportions of potential producers, for both the body impressions and the associated tracks, following a synapomorphy-based approach and considering also biostratigraphic and paleobiogeographic distributions.^23^

### Quantification and statistical analysis

#### Skeletal and trace fossil measurements

Footprint and skeletal measurements were listed in the text.

#### Ancestral state reconstruction

The ancestral state reconstruction was performed using the statistical analysis software R^24^ and the package phytools.^25^ The ancestral state reconstruction was run using the ER model. All the data used for the ancestral state reconstruction is listed in Table S1.

## Supplemental information

Table S1. Database of cloacae external morphology
